# Isolated Male Epispadias: Anatomic Functional Restoration Is the Primary Goal

**DOI:** 10.1155/2016/6983109

**Published:** 2016-09-18

**Authors:** Anne-Francoise Spinoit, Tom Claeys, Elke Bruneel, Achilles Ploumidis, Erik Van Laecke, Piet Hoebeke

**Affiliations:** Department of Urology, Ghent University Hospital, Ghent, Belgium

## Abstract

*Background*. Isolated male epispadias (IME) is a rare congenital penile malformation, as often part of bladder-exstrophy-epispadias complex (BEEC). In its isolated presentation, it consists in a defect of the dorsal aspect of the penis, leaving the urethral plate open. Occurrence of urinary incontinence is related to the degree of dorsal displacement of the meatus and the underlying underdevelopment of the urethral sphincter. The technique for primary IME reconstruction, based on anatomic restoration of the urethra and bladder neck, is here illustrated.* Patients and Methods*. A retrospective database was created with patients who underwent primary IME repair between June 1998 and February 2014. Intraoperative variables, postoperative complications, and outcomes were assessed. A descriptive statistical analysis was performed.* Results and Limitations*. Eight patients underwent primary repair, with penopubic epispadias (PPE) in 3, penile epispadias (PE) in 2, and glandular epispadias (GE) in 3. Median age at surgery was 13.0 months [7–47]; median follow-up was 52 months [9–120]. Complications requiring further surgery were reported in two patients, while further esthetic surgeries were required in 4 patients.* Conclusion*. Anatomical restoration in primary IME is safe and effective, with acceptable results given the initial pathology.

## 1. Introduction

Isolated male epispadias (IME) in the absence of bladder-exstrophy-epispadias complex (BEEC) is a rare malformation, with an estimated incidence in Europe around 0.6 per 100 000 live male births [1–3]. Like hypospadias, it covers a wide spectrum in which the meatal orifice can be located anywhere from the distal penile shaft to the pubic area. Unlike in hypospadias, the severity of the condition is related not only to the meatal position but also to the degree of incontinence associated with the meatal position, as the bladder neck might be involved in more proximal variants of IME [3, 4]. The goals of different reconstruction techniques are to provide continence in the proximal variants of the condition while achieving a cosmetically acceptable penile appearance. Different techniques have been described, from total penile disassembly to staged repair without disassembly [1, 2, 5, 6]. When discussing continence, IME is often considered together with BEEC. However, even if sphincter insufficiency is often observed in the most proximal forms of IME, certain children possess some degree of outlet competence, in contrast to those with a BEEC condition [7]. Restoring a normal anatomy will reapproximate the potentially present sphincter mechanisms and might therefore be sufficient in some children to warrant continence without further bladder outlet reconstruction. The technique of reconstruction of IME as restoration of a normal anatomy without any additional bladder outlet reconstruction is illustrated and evaluated in this manuscript.

## 2. Methods and Patients

### 2.1. Study Population

Data were collected retrospectively from the medical records of a consecutive series of 26 patients operated on for primary epispadias repair in a tertiary reference centre between 06/1998 and 02/2014. Male patients who underwent primary epispadias repair were selected. All patients' legal guardians were counselled about the risks and benefits of the treatments and signed an informed consent. This study protocol was approved by the local ethical committee at the Ghent University Hospital. Preoperative evaluation included medical history and physical examination. A retrospective database was created and descriptive statistics were reported using IBM SPSS Statistics for Windows, Version 22.0, IBM Corp., Armonk, NY.

Patients were classified according to the severity of their condition: penopubic epispadias (PPE) for the most proximal conditions, with or without open bladder neck, penile epispadias (PE), or glandular epispadias (GE) for the most distal variants.

All patients underwent the same standardized technique, with systematic postoperative drip stent in Cavi Care® (Smith and Nephew, Hull, United Kingdom) foam for 7 days postoperatively. Oral oxybutynin according to the child's weight was systematically administered to prevent bladder spasms. Systematic removal of the foam Cavi Care dressing was performed at day 7 in the outpatient clinic. A postoperative evaluation was planned 3 months after surgery and then on an annual basis.

### 2.2. Technique

Surgery was performed under general anesthesia, with additional caudal analgesia. Surgeons used 2.5x magnifying loupes during the procedures. Preoperative antibiotics (Cefazolin, according to child's weight, one shot) were administered before incision. The child was placed in a supine position, carefully padded to avoid compression points. A thermal probe was inserted in the rectum allowing monitoring during surgery.

The same standardized technique was applied to all variants of IME, regardless of the severity of the condition. Aim of the technique is anatomic restoration of the structures, without any additional intent of bladder neck reconstruction in case of PPE. GE involves only the distal portion of the urethra at the level of the splayed glans, with minimal outward rotation of the corpora cavernosa and a variable foreskin. In PE, foreskin is deficient and the meatus opens on the dorsal aspect of the penile shaft below the splayed glans corona. Varying degrees of curvature are observed. In PPE, the urethral opening is at the level of the penopubic junction, the entire urethra is open up to the bladder neck, and the penis is dorsally curved and shortened. In case of GE or PE, the technique was limited to penile reconstruction, without bladder neck reapproximation. Regardless of the technique, the main issues to be addressed in epispadias reconstruction are correction of chordee and skin closure, achievement of a cosmetically acceptable result, urethral reconstruction to allow micturition and ejaculation, and glanular reconstruction while preserving the genital sensitivity, leaving the neurovascular bundles unharmed.

All procedures started with placing a stay suture (Prolene 4/0) in the glans for traction.

In case of PPE, an initial suprapubic incision was realized, allowing bladder neck reapproximation after release of the surrounding tissues. This incision is realized in the line of the circumcision incision realized in PE and GE, to allow tension-free closure of the defect extending to the pubic area. The bladder was closed in 2 layers with monofilament sutures 4/0, without any additional attempt to tighten or recalibrate the bladder neck.

**Figure 1:**
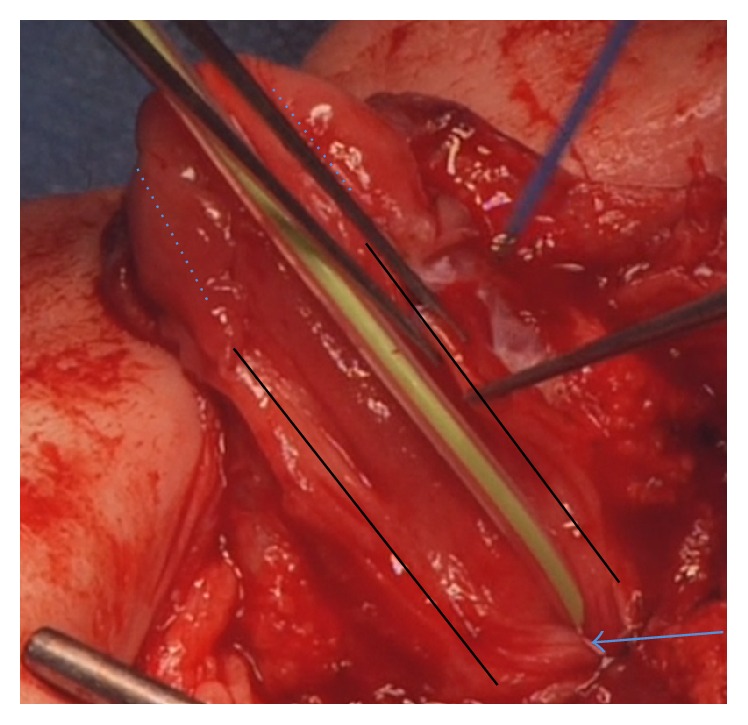
Mobilization of the urethral plate to allow tubularization. The arrow indicates urethral orifice. The black lines indicate the borders of the mobilized urethral plate. The dotted lines indicate the line of incision of the glans for development of glans wings.

**Figure 2:**
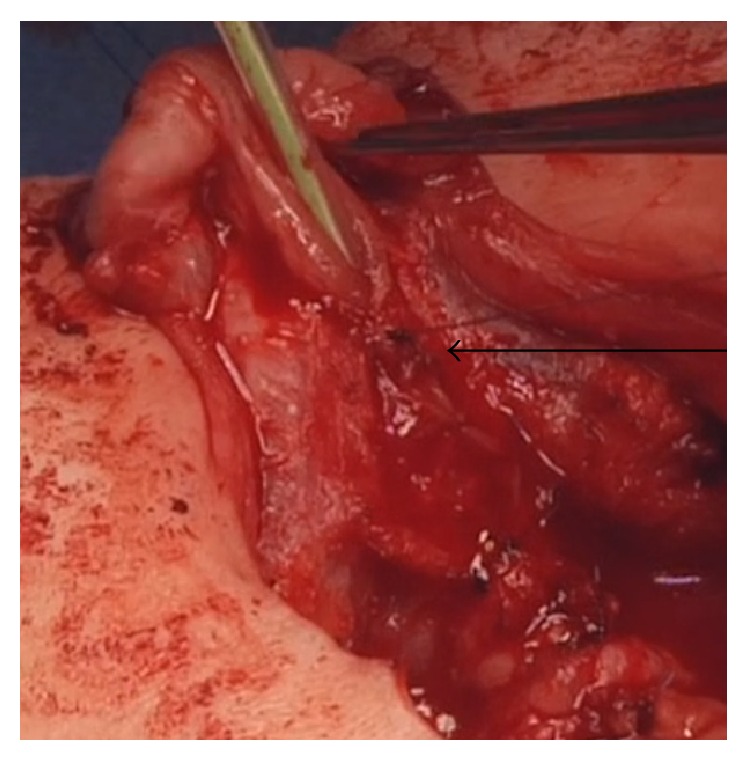
The figure shows the urethral plate halfway tubularized (arrow). The glans wings are developed to allow further closure of the urethra into the glans.

**Figure 3:**
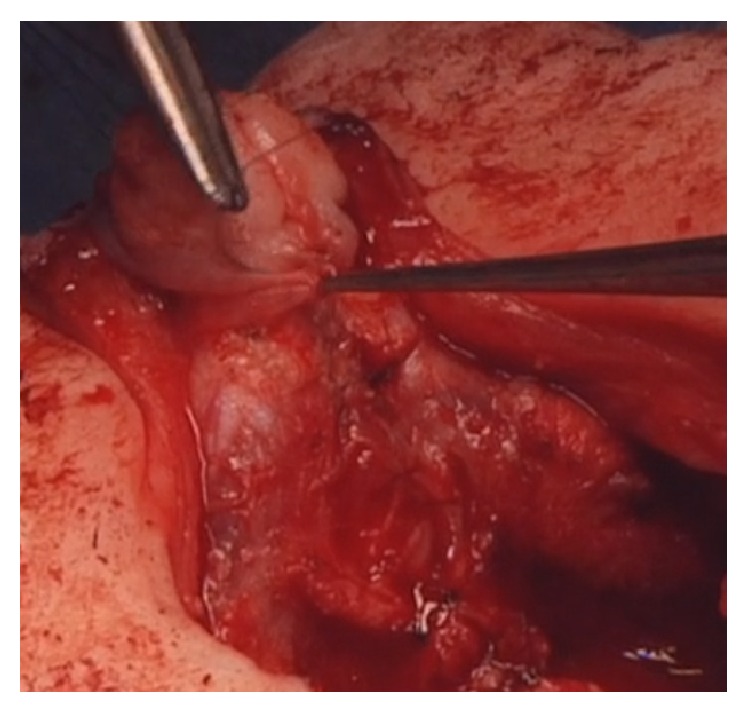
Closure of the glans over the neourethra in the glans and reconstruction of the glans.

The rest of the procedure focused on the penile and glandulae reconstruction. The technique might be considered as a mirror image of the Thiersch-Duplay principle of tubularization of the urethral plate [8]: after circumcision incision of the ventral aspect of the penis, the penis was degloved, thereby releasing dorsal chordee. Complete mobilization of the urethral plate was achieved to allow tubularization of the urethra toward the glans (Figure 1). The urethra was closed on a 10-French drip stent with a single submucosal running suture. Glans wings were developed to allow closure of the glans over the neourethra (Figures 2 and 3). Minimal endorotation of both corpora cavernosa was achieved, staying at large from the neurovascular bundle, to restore the shape of the glans and achieve an acorn-like aspect. No complete disassembly was performed in an effort to minimize the invasiveness of the surgery. By performing this endorotation of the corpora, the reconstructed urethra could be restored in a more anatomical position. A dartos tissue layer was brought on the neourethra as a waterproofing layer. The skin was closed with monofilament sutures.

A video of our technique can be found in Supplementary Material available online at http://dx.doi.org/10.1155/2016/6983109.

#### 2.2.1. Postoperative Course

Patients were admitted for the surgery in a day clinic and discharged the same day. They went home with the foam dressing and the drip stent in a double diaper system. They were provided with antibiotic prophylaxis and oral oxybutynin. They came back to the outpatient clinic for removal of the drip stent and the foam at day 7 after surgery.

#### 2.2.2. Postoperative Follow-Up

Follow-up is scheduled 3 months after surgery for a clinical check-up and subsequently on an annual basis.

#### 2.2.3. Data Analysis

Demographic data, pre- and perioperative variables, and follow-up variables were extracted from medical files and recorded in a dedicated database. Descriptive statistics were carried out for the available variables. Categorical variables were reported as frequencies and percentages and continuous variables as median and interquartile ranges (IQRs).

## 3. Results

Between June 1998 and February 2014, 26 consecutive patients were found that underwent IME repair. After exclusion of cripple and/or redo cases, 8 primary IME cases were selected for further analysis. Median age at surgery was 13.0 months [7–47]. Median follow-up time was 52 months [9–120]. No early complications within the 7 days before dressing and catheter removal were observed. Complications requiring further surgery were reported in two patients (fistulae in both patients presenting initially PPE and resection of urethra diverticulum in one patient with PPE), while further esthetic surgeries were required in 4 patients (excessive skin locally). Of those patients, all are continent, without urinary leakage or the need for continence pads, except for one patient with an initial PPE with open bladder neck. One patient presenting a GP has not yet reached a suitable age for potty-training and needs to be followed up further to evaluate his continence.

## 4. Discussion

Many techniques are reported for BEEC reconstruction, with variable success rates. However, the literature on IME is very scarce, and continence as an outcome is even less reported [4, 9, 10]. Most series report populations including BEEC and IME, making it very difficult to assess the specific outcomes of IME as a distinct congenital penile malformation [11].

Traditional management in IME, based on expert opinions, consists of IME repair in the first year of life. A few techniques are described, based on Cantwell's initial description of a technique where the corpora are mobilized and the urethra is placed in a hypospadic position [6]. It was updated by Ransley who described the incision of the corpora and their dorsomedial anastomosis above the urethra, leading to the classical Cantwell-Ransley modern technique for epispadias management [12]. Mitchell and Bägli pushed the technique further, introducing the complete disassembly concept by detaching the urethral plate not only from the corpora but also from the glans [13]. Complete disassembly is indeed traditionally performed in BEEC complex by numerous surgeons, but whether such an invasive procedure is necessary in IME has never been assessed. Our technique aimed at a minimally invasive reconstruction and postulates that if sphincter mechanisms are present, an anatomical restoration of the existing structures might be sufficient. Of our 8 patients, 6 are continent without additional need for continence procedures, and one patient has not yet reached age for continence evaluation. In comparison, the literature reports continence rates around 25%, even considering mild epispadias where only penile shaft is involved [1, 4, 9].

Urodynamic study in our incontinent patient showed intrinsic sphincter insufficiency. Additional surgery is planned to acquire continence.

These series of patients are too small to be able to state that a minimal invasive approach for IME reconstruction is enough. Further follow-up of patients and inclusion of a higher number of patients are needed to reach clear conclusions. However, given the invasiveness of the “gold standard” with complete disassembly, a minimal approach as described here gives promising results, without compromising further additional surgeries if needed.

## 5. Conclusion

Anatomical restoration in primary IME is safe and effective, with acceptable results given the initial pathology. Anatomical restoration might be sufficient in IME to acquire continence, but inclusion of a larger series of patients is needed to support this statement.

## Supplementary Material

This video showd isolated male epspadias reconstruction in a peno-pubic epispadias patient.

## Abbreviations

IME: Isolated male epispadias
BEEC: Bladder-exstrophy-epispadias complex
PPE: Penopubic epispadias
PE: Penile epispadias
GE: Glandular epispadias
IQR: Interquartile range.

## References

[1] Duckett J. W. (1977). Use of paraexstrophy skin pedicle grafts for correction of exstrophy and epispadias repair. *Birth Defects Original Article Series*.

[2] Perovic S., Scepanovic D., Sremcevic D., Vukadinovic V. (1992). Epispadias surgery—Belgrade experience. *British Journal of Urology*.

[3] Gearhart J. P., Leonard M. P., Burgers J. K., Jeffs R. D. (1992). The Cantwell-Ransley technique for repair of epispadias. *Journal of Urology*.

[4] Kaefer M., Andler R., Bauer S. B., Hendren W. H., Diamond D. A., Retik A. B. (1999). Urodynamic findings in children with isolated epispadias. *The Journal of Urology*.

[5] Dees J. E. (1949). Congenital epispadias with incontinence. *The Journal of Urology*.

[6] Cantwell F. V. I. (1895). Operative treatment of epispadias by transplantation of the urethra. *Annals of Surgery*.

[7] Braga L. H. P., Lorenzo A. J., Bägli D. J., Khoury A. E., Pippi Salle J. L. (2008). Outcome analysis of isolated male epispadias: single center experience with 33 cases. *The Journal of Urology*.

[8] Subramaniam R., Spinoit A. F., Hoebeke P. (2011). Hypospadias repair: an overview of the actual techniques. *Seminars in Plastic Surgery*.

[9] Kramer S. A., Kelalis P. P. (1982). Assessment of urinary continence in epispadias: review of 94 patients. *The Journal of Urology*.

[10] Higuchi T., Holmdahl G., Kaefer M. (2016). International consultation on urological diseases: congenital anomalies of the genitalia in adolescence. *Urology*.

[11] Carrasco A., Vemulakonda V. M. (2016). Managing adult urinary incontinence from the congenitally incompetent bladder outlet. *Current Opinion in Urology*.

[12] Kajbafzadeh A. M., Duffy P. G., Ransley P. G. (1995). The evolution of penile reconstruction in epispadias repair: a report of 180 cases. *The Journal of Urology*.

[13] Mitchell M. E., Bägli D. J. (1996). Complete penile disassembly for epispadias repair: the mitchell technique. *The Journal of Urology*.

